# Effect of PET Size, Content and Mixing Process on the Rheological Characteristics of Flexible Pavement

**DOI:** 10.3390/ma15103565

**Published:** 2022-05-16

**Authors:** Teyba Wedajo Mahdi, Sanjaya Senadheera, Tewodros Ghebrab

**Affiliations:** 1 Department of Transportation Engineering, Jimma Institute of Technology, Jimma University, Jimma P.O. Box 378, Ethiopia; 2 Department of Civil, Environmental and Construction Engineering, Texas Tech University, Lubbock, TX 79409, USA; sanjaya.senadheera@ttu.edu (S.S.); tewodros.ghebrab@ttu.edu (T.G.)

**Keywords:** asphalt, binder, PET, pavement, plastic waste, bitumen

## Abstract

The performance of asphalt binder reinforced with waste plastic polyethylene terephthalate (PET) was investigated. Penetration, ductility, softening point, and rotational viscosity tests were conducted to check the performance of the PET-reinforced pavement. The rheological properties of the binder were determined using amplitude sweep and frequency sweep tests and performance grade (PG) measurements of aged and unaged specimens. PET size, mix mechanism, and mix temperature significantly influenced the physical properties of the AB and the penetration index (*PI*). The size and content of PET had pronounced effects on the *PI* and softening point than the blending temperature. Increasing the size of PET particles from 75 to 150 μm and the content from 0% to 10% of the bitumen resulted in the reduction of the penetration and ductility values from 96 to 85 mm and 100 to 78 cm, respectively, whereas the softening point increased from 46 to 56.6 °C. As a result, the *PI* value of the binder increased, which indicates that the temperature susceptibility was improved. The addition of 10% PET increased the viscosity of the baseline bitumen by threefold upto a temperature of 135 °C and dropped it by fourfold when the temperature was raised to 165 °C. Increasing the PET from 0% to 10% and the temperature from 21.1 to 54.4 °C increased the critical strain value (LVER) by 96%.

## 1. Introduction

Asphalt binder is widely used as a pavement material for the upper layer of the road surface. It has been extensively used for its benefits of binding aggregate, reducing noise pollution and providing a smooth and comfortable ride [1,2,3]. However, bitumen on the road surface ages due to oxidation [4], loses its ductility, and becomes susceptible to cracking. Asphalt binder ages rapidly exposed to the physical and chemical factors such as extreme temperature, volatility, oxidation, decomposition, and polymerization, which change the structure and chemical composition of the asphalt [5,6,7,8,9,10,11,12,13] and weaken the pavement.

Asphalt needs to be thicker to have durable and aging-resistant pavement. However, this demands significant material resources and construction costs and could cause the depletion of nonrenewable resources. PET has chemical and physical properties capable of withstanding the extreme environment that can damage the integrity and performance of the flexible pavement. Therefore, PET needs to be investigated to be used as potential supplementary asphalt material. The steady population growth has increased the use of plastic materials, causing solid waste management problems, a serious global challenge, especially in developing countries [14,15,16,17].

One of the most viable solutions to minimizing PET waste management problems and improving asphalt pavement performance is using PET as a partial replacement for asphalt binder [18,19,20,21,22,23].

On the other hand, using plastic waste as a partial replacement for asphalt binder improves pavement performance and reduces economic and environmental concerns and reliance on natural resources [9,13,24,25,26].

PET bottles can be used as alternative solutions in road construction projects to minimize environmental pollution due to waste plastics and the depletion of natural resources. Recently, many researchers [27,28,29,30,31,32,33,34,35,36,37,38,39,40,41,42] have looked for alternative products or ways of using different waste plastics, such as crumb rubber [27,28,29,30,31], polyethylene and polypropylene [32,33,34], polyvinyl chloride [35,36,37], ethylene-vinyl acetate [38,39,40,41,42], and PET [40,41,42], to modify HMA, improve asphalt binder properties, and protect the environment.

Several comparative studies [43,44,45] focused on the effects of various waste plastic types and evaluated their ability to improve the performance of asphalt binders [43,44]. However, limited reports are available on the properties of PET-modified asphalt binder, which have either reported inconsistent results or missed investigating some essential aspects such as the effects of mixing process, particle size, and temperature variation on important consistency and rheological properties of the asphalt binder.

The objective of this study was to investigate the effect of partial replacement of asphalt binder by PET on the properties and performances of the binder. The changes in the consistency and rheology of the asphalt binder were investigated based on several parameters such as PET size and content and mixing mechanisms, which are dry and wet mix processes. The effects of each parameter on the properties of the PET-modified binder were determined using consistency, Superpave and rheological characteristics tests.

## 2. Materials and Methods

### 2.1. Bitumen

Bitumen asphalt binder with Pn 80/100 penetration grade, a widely used base binder in Ethiopia, was used in this study. The properties of the bitumen determined in the laboratory are shown in Table 1.

### 2.2. Polyethylene Terephthalate Plastics Fiber

**Figure 1:**
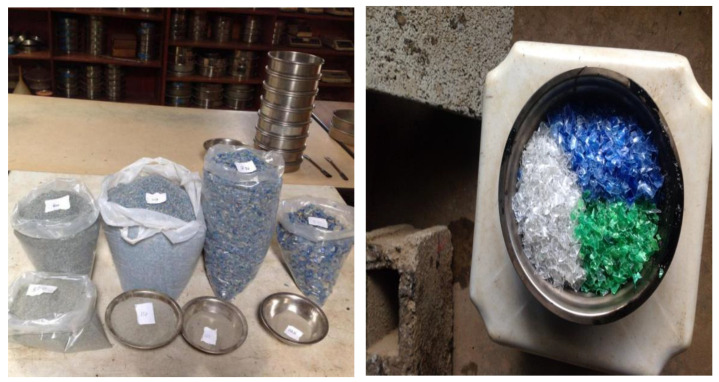
Coarse and fine PET particles.

Commercially available waste polyethylene terephthalate (PET) was obtained from the Roha Pack PLC in Addis Ababa, Ethiopia. The PET was collected, classified according to type and color, shredded, dried in an oven, and crushed into finer particles using PS-900 plastic bottle crushing machine, as shown in Figure 1. Two PET particle sizes were used: particles finer than 75 μm and those between 75 μm and 150 μm in size. The properties of the PET are listed in Table 2.

### 2.3. Selection and Mixing Mechanism of PET-Modified Asphalt Binder

The PET particles were added to the asphalt binder following the procedures reported in the literature [9,25,46,47,48]. The concentrations used were 0%, 3%, 5%, and 10% by weight of the binder. Previous studies [47,48,49] have utilized fine and coarse graded PET particles in various size ranges in asphalt mixture modification. In this study, two size ranges of PET were used, namely 150 to 75 µm, for the consistency test, and 75 µm was used for the rheological test.

### 2.4. Preparation of PET Modified Binder for Consistency Test

Two sample groups of wet-mix (WM) and dry-mix (DM) processes were prepared using the two PET particle size ranges. In the DM process, the bitumen was heated to 150 °C until it became liquid asphalt and thoroughly mixed with the PET particles in the amount of 3%, 5%, and 10% by weight of the binder, maintaining a constant weight of the PET-modified binder. Three replicate samples were prepared for each combination of PET size, content, and mix mechanisms at a constant temperature. A total of 48 samples were prepared to test the physical properties of the PET-modified bitumen. The penetration index (PI) and ductility values of the modified binder were determined from the softening point (ASTM D36) and penetration (ASTM D5) test results. A rotational viscosity test was performed on the binder, following ASTM D4402 standard procedures, at 135 and 165 °C, and the average results for all samples are reported in the following sections. 

In the WM process, the PET-modified bitumen was heated to 150 °C and the PET until it melted between 250 °C to 300 °C. A sufficient quantity of the heated bitumen and PET were blended using a blender at 600 rpm for 30 minutes at an average temperature of 250 °C. The difficult part was keeping the mix temperature constant and blending the PET particles with the bitumen. The melted PET immediately became stiff and brittle. According to Fang et al. [9] and Abdullah et al. [50], the WM process is not feasible for plastics such as PET because it is extremely difficult to achieve a uniform blend and avoid segregation, leading to more oxidation at high temperatures. Thus, the WM process was dropped as an option. 

### 2.5. Sample Preparation of PET Modified Binder for Rheological Test

Before conducting the rheological test, consistency tests were considered in selecting the PET particle size and mix mechanism, which resulted in the selection of the 75 μm size PET particles and dry-mix process. 

Two types of samples were prepared for the aging test: original and aged binders. The aging process utilized in the study was the rolling thin film oven (RTFO). The percentages by weight replacement of PET for bitumen were 3%, 5%, and 10%, and the mix temperature was kept constant at 150 °C for all modified and unmodified asphalt binders.

### 2.6. Rheological Characterization of Asphalt Binder

#### 2.6.1. Amplitude Sweep Test Result

An amplitude sweep test was performed to determine the complex modulus and phase angle values for the shear strain response and the asphalt binder’s linear viscoelastic region (LVER). The test was carried out at a constant frequency of 10 rad/s with a minimum shear stress of 100 Pa and maximum shear stress of 90,000 Pa based on the AASHTO T315 standard procedure. LVER was used to measure damage according to the AASHTO TP 101-12 standard procedure, which was applied to evaluate the fatigue resistance of the binders. Amplitude and frequency sweep tests were performed at intermediate temperatures of 54.4, 37.8, and 21.1 °C. Performance grade (PG) test temperatures were based on a maximum temperature of 50 °C in Dallol (Afar, Ethiopia) and the criteria specified in SHRP-A-410.

#### 2.6.2. PG Determination

Standard procedures were used to determine the PG of an unknown asphalt binder or verify the PG of a known asphalt binder. According to AASHTO T 315 standard test procedure, the original binder at 12% strain and the RTFO aged sample at 10% strain were used for both unmodified and modified binders. Following the AASHTO M320 test procedure, the samples were subjected to an initial temperature of 58 °C and gradually increased by six degrees up to 70 °C. The original binder’s rutting parameter (G*/sinδ) should exceed 1 kPa and 2.2 kPa for the unaged and aged binders, respectively.

#### 2.6.3. Frequency Sweep Test

The frequency sweep test was used to assess the rheology of binder deformation and flow under different loading conditions in terms of the basic rheological parameters, such as shear modulus (G*) and phase angle (δ). A strain value of 1% was used because strain values less than 1% were close to the nonlinear viscoelastic region. Frequency sweeps or variations were set from high to low frequency (25 to 0.1 Hz) at 21.1, 37.8, and 54.4 °C, increasing the damaging effect on all aged and unaged binders samples. A master curve was created using the principle of time and temperature superposition [51]. Changes in complex modulus and phase angle were investigated using a reference temperature of 21.1 °C.

## 3. Results

### 3.1. Convection Test Result

#### 3.1.1. Effect of Size and Content of PET on *PI* Value

Waste plastic can improve the performance of bitumen and its temperature sensitivity. The convectional bitumen characteristics such as penetration, softening, and ductility cannot adequately determine the temperature sensitivity of PET-modified or unmodified bitumen. The conformity of bitumen was determined using the *PI* value, a quantitative measure of the response of a binder to the variation in temperature sensitivity, and is defined as follows [52].

(1) $$PI=\frac{\left(1952-500\right)\;\times \;(\log Pn_{25})-20\;\times \;Sp}{50\;\times \;\log Pn_{25}-Sp-120}$$

where $Pn_{25}$ is penetration at 25 °C, Sp is softening point temperature (in °C) of the PET-modified and unmodified binder, and *PI* is penetration index with values ranging between −1 and +1. The effect of PET content and size on the *PI* values of bitumen are summarized in Table 3. 

Table 3 lists the *PI* values for unmodified and modified bitumen with two sizes of PET (75 and 150 μm). The results indicate that increasing the PET content and size increased the softening temperature and decreased the penetration value, corresponding to an increase in the *PI* value. The *PI* value increased from −0.611 to 1.6687 and −0.611 to 1.2692 for PET sizes of 150 and 75 μm, respectively, when the PET content increased from 0% to 10%. Thus, increasing the percentage replacement of bitumen by PET from 0% to 10% has increased the *PI* value by 308% and 373%, respectively, for the 75 and 150 μm PET particle sizes. As shown in Table 3, the increase in the size and amount of PET increased the softening point due to the blending temperature effect and physical mix. Conversely, the penetration value decreased as PET made the bitumen stiffer. This caused the bitumen to have higher *PI* values. A higher *PI* value indicates an improvement in temperature susceptibility, making higher resistance to temperature crack and deformation.

#### 3.1.2. Effect of Mix Process on Physical Properties of Asphalt Binder

Table 4 shows the effect of mixing mechanisms (wet and dry mixing process) on the basic empirical priorities of PET-modified and unmodified bitumen, with different PET content and size. The results in the dry mix indicate that an increase in PET size from 75 to 150 µm and content from 0% to 10% has lowered the penetration and ductility values while increasing the softening point. This could be attributed to the advantage of the dry process, which modifies the binder properties that act as a lubricating agent, allowing PET particles to make bitumen stiffer (decrease penetration) and harder (increase the softening point). Thus, an increase in the content and size of PET in the base binder using a DM process mechanism caused a higher softening point and poor elongation or decreased interlocking of PET molecules with the bitumen, which decreased the ductility value. It was difficult to apply the WM process to blend the PET with bitumen. PET is a thermosetting material that hardens when heated to a temperature higher than 250 °C, making it impossible to mix with hot bitumen. The results of the WM process test are summarized in Table 4, where all the above empirical test results remain constant and the same as the baseline bitumen result. It may be due to the PET particles not melting at the stated temperature. According to Asphalt Institute Technical Advisory Committee, a modified asphalt binder using a WM process is impossible or impractical.

#### 3.1.3. Effect of Mixing Temperature on Physical Properties of Asphalt Binder

Determining the blending temperature by modified bitumen helped determine the performance and conformity, which is a vital criterion for production and using PET-modified asphalt binder.

**Figure 2:**
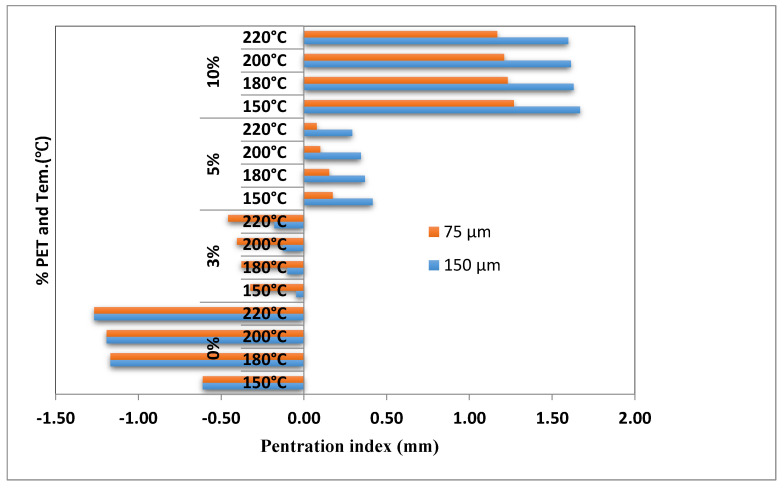
PET content, Tem vs. *PI* value of asphalt binder.

As shown in Figure 2, the *PI* values are out of range (−1 and +1) when the blending temperature was >150 °C on unmodified bitumen and the 10% PET content bitumen. The result implies that the *PI* value of bitumen is directly proportional to the PET content and size rather than the blending temperature. It can be concluded from the results that the stated mix temperature is not necessarily sufficient to dissolve the PET particle. Thus, the viable option to improve and bring the desirable physical proprieties of bitumen is to use large size but low content PET or vice versa. In line with the above findings, numerous studies have reported that the content and size of modifiers have more significant effects on modified bitumen than the mix temperature and blending time [9,49,53].

As per the results summarized in Table 4, the blending temperature and PET content and size have more pronounced effects on the penetration and softening points than the ductility values. For instance, as indicated in the above table, at a constant temperature of 150 °C, the variation in PET from 0% to 10% of bitumen resulted in the decline of the penetration and ductility values from 96 to 85 mm and 100 to 78 cm, respectively, but the softening point increased from 46 to 56.6 °C.

On the other hand, inconsistent results were observed for binders containing 150 µm size 10% PET. For instance, when the temperature was increased from 150 to 200 °C, the penetration value increased from 85 to 85.6 mm; the softening point decreased from 56 to 55.6 °C; and the ductility values showed no change, remaining 78 cm.

### 3.2. Rheological Properties of Rubberized Bitumen

#### 3.2.1. Effect of Mix Process Technique on Viscosity of PET Modified Asphalt Binder

**Figure 3:**
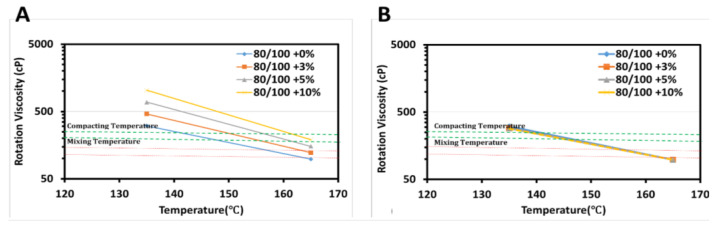
Viscosity-temperature curve for 75 µm PET: (**A**) dry mix mechanism, (**B**) wet mix mechanism.

**Figure 4:**
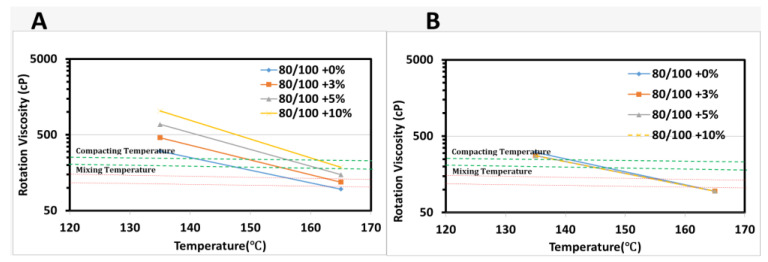
Viscosity-temperature curve for 150 µm PET: (**A**) dry mix mechanism, (**B**) wet mix mechanism.

Figure 3 and Figure 4 summarize the viscosity results of the unmodified and modified binders at temperature settings of 135 and 165 °C with different sizes and content of PET applying two mixes (dry and wet) processes. The viscosity of baseline bitumen was 306 and 98 cP under 135 and 165 °C, respectively, which was less viscose than the 10% PET content bitumen at 1032 and 190 cp (Table 4). The mixing and compaction temperatures are important parameters in viscosity analysis and are determined from the log-log graph. They were out of range when incorporating 10% of PET to baseline bitumen for the DM process, but asphalt binder did not ensure the ability to mix properly for all percentage levels of PET adopted to wet process. The result shows that the viscosity value was decreased with an increase in the PET size from 75 to 150 µm and the temperature from 135 to 165 °C. This cloud be due to the large size of PET particles being insoluble at the stated temperatures, which made the bitumen stiffer and decreased the ability to pump. When applying the DM process, the viscosity increased with the PET content. It was also observed that the addition of 10% PET increased the viscosity value three times that of baseline bitumen under 135 °C. On the other side, the viscosity value declined up to four times when the temperature increased from 135 to 165 °C at 10% PET. All viscosity values recorded were within the acceptable range of the specified limit (3000 cP). These findings agree with those reported by other researchers [9,25,54,55], justifying that increasing the waste plastic content significantly increased the viscosity, under constant and different temperature. This indicates the increase of viscosity assured the binder’s workability and the bitumen’s ability to be pumped and coat the aggregate properly.

#### 3.2.2. Effect of PET Content, Temperature, and Aging on Binder LVER

**Figure 5:**
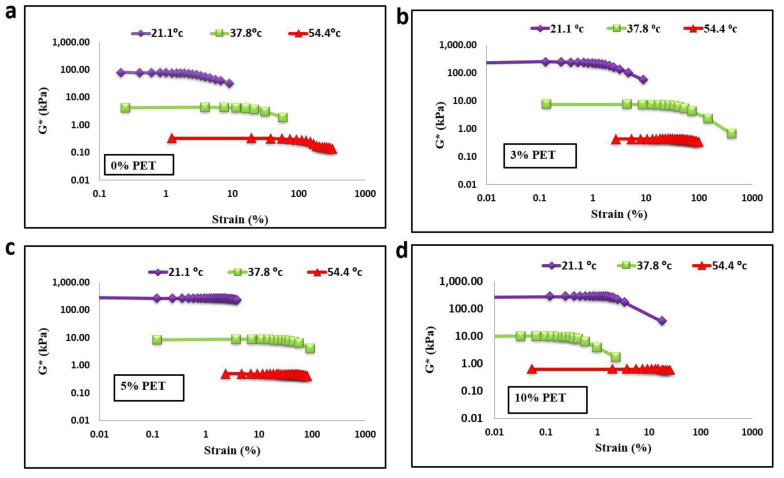
Linear viscoelastic range of unaged binder with PET content of (**a**) 0%, (**b**) 3%, (**c**) 5%, and (**d**) 10%.

**Figure 6:**
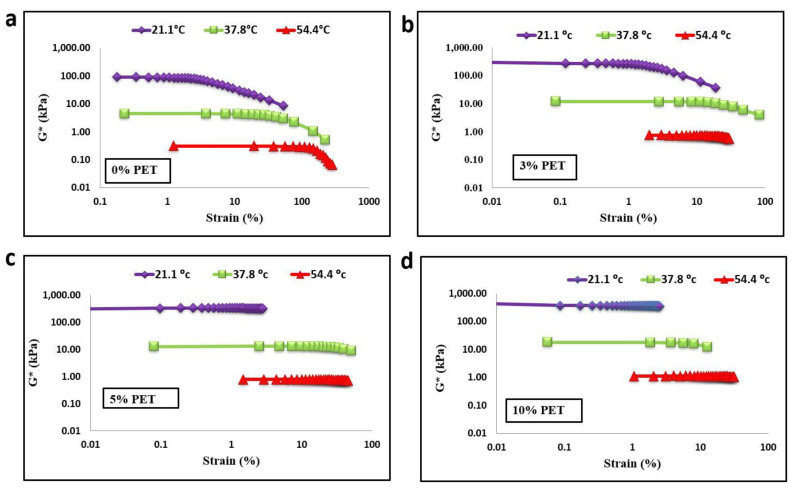
Linear viscoelastic range of RTFO-aged binder with PET content of (**a**) 0%, (**b**) 3%, (**c**) 5%, and (**d**) 10%.

As shown in Figure 5 and Figure 6, temperature, modifiers, and aging were considered during the amplitude sweep test to check the strain response and change in stiffness, which affect the linear viscoelastic region (LVER) in the bitumen. Increasing the PET content to 10% of bitumen increased the LVER more than that of the unmodified bitumen for both aging statuses. It showed that the PET particle modifiers had a significant effect on the rheological properties of the asphalt binder. In terms of the aging condition, it is evident that the LVER of the unaged sample was less than that of the RTFO-aged samples in both modified and unmodified bitumen. As the temperature increased, the LVER of the RTFO-aged unmodified and modified binder increased. For example, as the temperature increased from 21.1 to 54.4 °C at 10% PET content, the LVER of RTFO-aged bitumen increased from 18% to 96%. This dynamic improvement in the LVER might be due to the aging process of PET-modified bitumen. This could be due to PET particles preventing bitumen from oxidizing due to their low-molecular oil content, which could have impeded oxidation in the PET-modified bitumen. According to Ghavibazoo et al. [5], during the aging process, polymer modifiers produce small molecules to absorb the light oil of the asphalt, which can reduce the content of free radicals and improve the aging properties of the asphalt. Therefore, it can be concluded that PET modifier has a positive effect on the value of LVER. This higher value of LVER improved the damage resistance as the binder could resist fatigue damage.

#### 3.2.3. PG Determination

Table 5 summarizes the effect of PET particle content and aging status on the properties of asphalt binder. The result shows that increasing the PET content of the baseline bitumen significantly increased the basic rheological properties such as complex shear modulus (G*) and stiffness and decreased the phase angle for all samples. This means the PET additive significantly improved the elastic modules of bitumen compared to the unmodified one. As shown in the above table, the value of PG was improved from PG 58 to PG 64 when 10% PET was added to the baseline bitumen. Improving the PG of a material predicts an improved rutting potential of asphalt binder. Additionally, the original and RTFO-aged PG values of the unmodified and PET-modified binders were compared. PG value of the RTFO-aged PET modified binder was higher than those of unaged modified and unmodified binders, which means PET additive significantly improved the RTFO aged binder compared to the unaged binder. In the super-pave test, stiffness and elasticity of material significantly influence the rutting performance of the material. Consequently, it can be concluded that PET modifier and aging status affect both asphalt stiffness and elasticity. It is more likely that higher stiffness (viscous) develops considerable stress against deformation and lower value of phase angle (more elasticity), enhancing the rutting and aging resistance of the material. The addition of modifiers had a positive effect on stiffens and elasticity. In this case, the modifiers had a greater effect on RTFO aged binder than the original binder [45,56,57,58].

#### 3.2.4. Frequency Sweep Test

**Figure 7:**
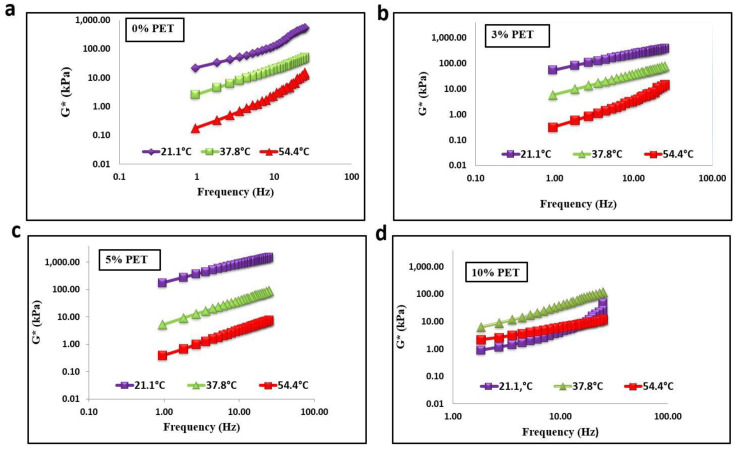
Isothermal plots of unaged binder with PET content of (**a**) 0%, (**b**) 3%, (**c**) 5%, and (**d**) 10%.

**Figure 8:**
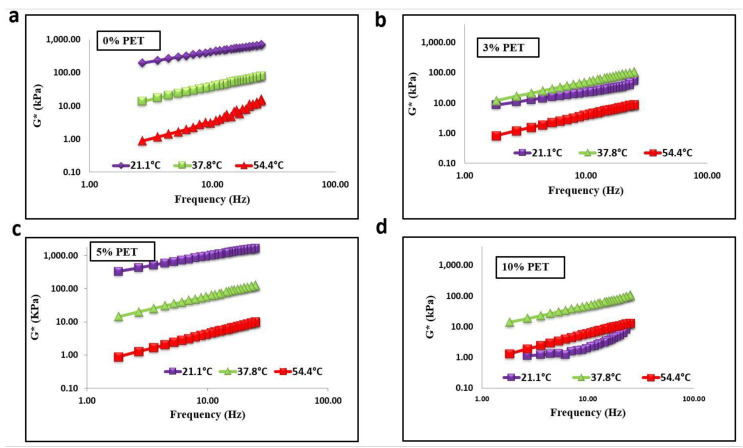
Isothermal plots of RTFO-aged binder with PET content of (**a**) 0%, (**b**) 3%, (**c**) 5%, and (**d**) 10%.

Figure 7 and Figure 8 show that, as the temperature increased, the shear stiffness decreased for all percentages of PET-modified binders; however, it increased with an increase in PET content. Contradictory results were observed when PET content exceeded 5% for aged and unaged binders. The waste plastic dosage greatly affected the rheological properties of asphalt binder [21]. The addition of PET to baseline bitumen significantly improved the shear modulus at high frequency rather than low frequency. The result also reveals that adding PET to baseline bitumen increased the proportion of elastic components in the asphalt binder. This means modifiers significantly improved the shear stuffiness at low frequency and high temperature than at low and high frequencies.

#### 3.2.5. Master Curves of Complex Shear Modulus

**Figure 9:**
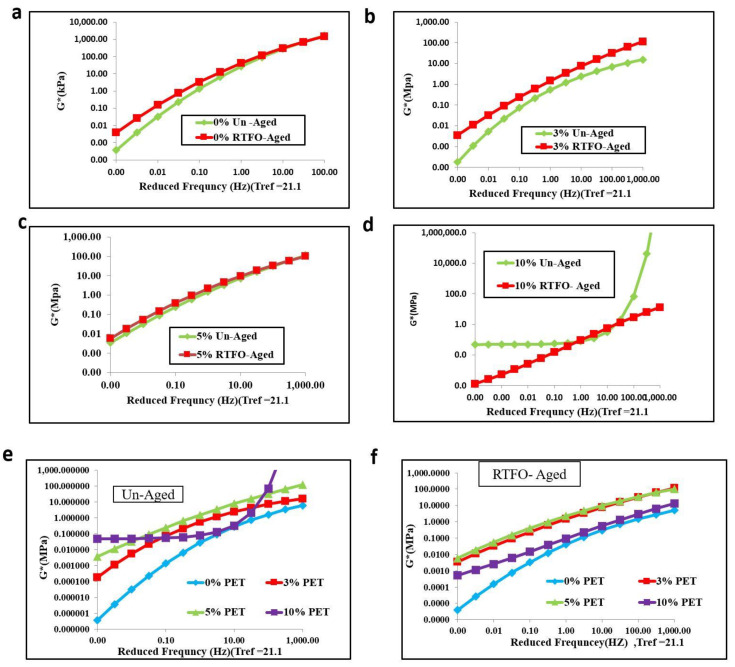
Master curve of complex modulus of unaged and RTFO-aged binder with PET content of (**a**) 0%, (**b**) 3%, (**c**) 5%, (**d**) 10%, (**e**) PET on unaged binder, and (**f**) PET on aged binder.

Figure 9 shows that the complex shear modulus (G*) behaved differently because of temperature, frequency, the content of the modifier, and aging status. It was observed that the G* increased gradually with the reduction of frequency up to 5% PET for both original and RTFO aged binder. At low frequency and high temperature, the modulus increased significantly with the modifier. However, the modifiers had no effect at high frequency and low temperature. It indicates that the modifier appreciably improved the complex shear modulus of the virgin binder at higher temperatures. This could be attributed to PET particles having improved the high-temperature performance of asphalt binder. In terms of aging condition, the maximum value of G* was observed at a low loading frequency of PET-modified asphalt binder. At high frequency, the sample structure showed disturbance when PET content exceeded 5% in an unaged binder, whereas the G* value declined more when incorporating 10% PET to RTFO aged bitumen than for the unmodified bitumen. This behavior was probably related to the aging properties of PET, which might negatively affect the stiffness of the asphalt binder. It may be concluded that the high-temperature performance of unmodified asphalt binder was improved after adding the PET particles. The effect of modifiers was more highly pronounced in the RTFO aged binder than in the unaged binder.

#### 3.2.6. Master Curves of Phase Angle

**Figure 10:**
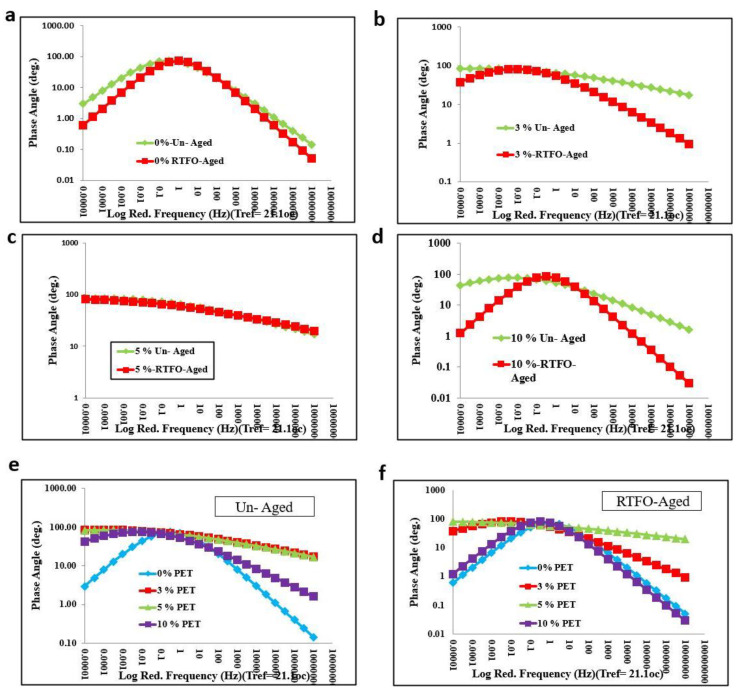
Master curve of phase angle of unaged and RTFO-aged binder with PET content of (**a**) 0%, (**b**) 3%, (**c**) 5%, (**d**) 10%, (**e**) PET on unaged binder, and (**f**) PET on aged binder.

The master curve phase angle displayed in Figure 10 represents the effect of PET content, temperature, and aging status on the phase angle. The phase increased steadily during the reduced frequency period from 0.00001 to 1 Hz when the PET content increased from 0% to 10% and decreased dramatically when the reduced frequency was higher than 1 Hz for both unaged and RTFO aged binder, in terms of the aging status effect on the phase angle. The phase angle of the unmodified binder was more sensitive to aging than PET-modified binders. Decreased phase angle indicated an increase in elastic modulus. The stable region of phase angle was observed at a high frequency and high percentage of PET (10%), which was due to PET particles forming a continuous elastic network during blending time with the bitumen, thus enhancing the material’s performance.

## 4. Conclusions

This study focused on experimental methods and the experiments were conducted using two approaches. The first approach was to evaluate the effect of PET additives on conventional tests such as penetration, ductility, softening point, and rotational viscosity tests by adopting dry and wet mix processes. The second approach was focused on the effect of PET particles on the performance parameter, which was accomplished by the DSR test (amplitude sweep, frequency sweep test, and PG) determination for RTFO aged and unaged material use in the DM process technique. Four different percentages of PET, 0%, 3%, 5%, and 10%, of 150 and 75 µm size were used for the initial approach. Based on the initial results, 75 µm size PET and DM process were selected for the second approach because the smallest size and dry mix process mechanism had good results for all tests. Finally, the following conclusions were drawn based on the analysis of the results.

- The properties of bitumen, such as penetration and softening point, were improved with the addition of the waste plastic PET fiber as compared to the baseline binder. The content and size of PET in bitumen had a significant effect, dramatically increasing the softening and decreasing the penetration value. Increasing the size of PET from 75 to 150 µm led the *PI* value to increase up to 31%, while the baseline bitumen accelerated four-fold when the PET content increased from 0% to 10%. The increase in the size and amount of PET increased softening point due to the blending of temperature effect and physical mix. The decrease in penetration value occurred as PET made the bitumen stiffer. This led the bitumen to have a higher *PI* value. A Higher *PI* value, in turn, implies the improvement of temperature susceptibility, which makes higher resistance to temperature crack and deformation.
- Rather than the blending temperature, the PET content and size had a pronounced effect on the penetration value and the softening point, while a constant ductility value was obtained. At constant 150 °C, a variation in PETcontent from 0% to 10% of bitumen resulted in a decline of the penetration and ductility values of the binder from 96 to 85 mm and 100 to 78 cm, respectively. The softening point increased from 46 to 56.6 °C when increasing the PET content from 0% to 10%. Utilizing PET particles improved the high-temperature susceptibility of the binder.
- The viscosity of baseline bitumen was 306 and 98 cP under 135 and 165 °C, which was less viscose than when 10% PET was incorporated into bitumen, reaching 1032 and 19 cP. Thus, adding 10% of PET increased the viscosity value three times the baseline bitumen under 135 °C. The viscosity value declined four times when the temperature increased from 135 to 165 °C at 10% PET.
- Modifiers had a greater effect at high temperatures than at low temperatures. PG results reveal that when 10% PET was added to the baseline bitumen, the PG value upgraded from PG 58 to PG 64. Additionally, compared to unaged and RTFO aged PG values, the PG value of RTFO aged was softer (not susceptible to hardening) than the unaged binders. The modifier significantly improved the shear stiffness and decreased phase angle (increase elastic modules) at low frequency and high temperature than at high frequency. With the help of PET additive, increasing the stiffness and elastic modulus of the binder the pavement performance was improved against this distress.

## Figures and Tables

**Table 1:** Asphalt Cement Quality Test Bitumen (80–100).

Tests	Results	Applicable Standard	Specification AASHTO M82
Penetration,100 g 5 sec at 25 °C	86	AASHTO T 49	80–100
Ductility, 5 cm/min, cm	100+	AASHTO T 51	min, 100
Specific Gravity of Asphalt, (g/mL)	1023	AASHTO T 228-06	1010–1050
Solubility in Trichlorethylene, %	99.8%	AASHTO T 44	min, 99.5
Loss On Heating	0.2%	AASHTO T 47	max, 0.2%
Softening Point	46.0 °C	AASHTO T 53	45–52 °C
Flash Point	588.2 0F	AASHTO T 48	min, 232 °C
Water Content	-	AASHTO T 55	max, 0.2%
Penetration of Residue	88.4	ASTM D5-IP49	min, 53
Ductility of Residue	100+	AASHTO T 51	min, 75

**Table 2:** Physical properties of waste PET [24].

Property	Result	ASTM Standard
density	0.9185 gm/cm^3^	D1,505,792
Melting point	265 °C	D3418
Thermal conductivity	0.15 to 0.24 W m^−1^ K^−1^	
Chemical unit	(CH2−CH2−)^−n^	
Flexural modules	0.203 GPa	D790
Tensile strength	10.11 N/mm^3^	D638

**Table 3:** Effect of PET content and size on *PI* values.

% of PET and Binder	Waste Plastic Pet Size					
	(150 μm)			(75 μm)		
	Av. Pen. @25 °C in mm	Av. Soft. °C	*PI* Value B/n [−1+1] 0.1 mm	Av. Pen. @25 °C in mm	Av. Soft. °C	*PI* Value B/n [−1+1] 0.1 mm
0%	96	46	−0.6110	96	46	−0.6110
3%	95	48	−0.0454	95.5	47	−0.3235
+5%	92	50	0.4163	93	49	0.1735
+10%	85	56	1.6687	87	54	1.2692

**Table 4:** Effect of mixing process on physical properties of asphalt binder.

% PET	Av. Pen@25 °C in mm		Av. Soft. °C		Ductility (cm)	
**Dry Mix Process**						
	(150 µm)	(75 µm)	(150 µm)	(75 µm)	(150 µm)	(75 µm)
0%	96	96	46	46	100	100
3%	95	95.5	48	47	89	90
5%	92	93	50	49	85	85
10%	85	87	56	54	75	78
**Wet Mix Process**						
0%	96	95.5	46	45	100	100
3%	96	95.5	46	45	100	100
5%	96	95.5	46	45	100	100
10%	96	95.5	46	45	100	100

**Table 5:** Performance Grade determination for baseline and PET modified binder.

Aging Status	PET %	Test Temp °C	G* (Pa) (×10^2^)	δ(o)	G*/sin delta @10rad/sec. Pa (×10^2^)	PG Pass Fail Tem (°C)	PG
**Super-pave Spec. Limit (kPa) > 1.0 kPa**							
Un-aged	0	58	14.9	85.7	14.9	61.7	PG 58-xx
		64	7.7	86.8	7.7		
		70	4.6	86.1	4.6		
	3	58	23.7	88.0	23.7	64.9	PG 64-xx
		64	10.7	88.7	10.7		
		70	4.9	88.8	4.9		
	5	58	27.2	87.7	27.3	65.7	PG 64-xx
		64	12.2	88.4	12.3		
		70	5.8	88.4	5.8		
	10	58	34.7	87.1	34.8	67.1	PG 64-xx
		64	14.7	87.7	14.7		
		70	7.0	87.7	7.0		
**Super-pave Spec. Limit (kPa) > 2.2 kPa**							
RTFO-aged	0	58	26.3	84.7	26.3	59.3	PG 58-xx
		64	11.7	86.4	11.7		
	3	58	39.6	85.9	39.7	62.3	PG 58-xx
		64	17.3	87.0	17.3		
	5	58	45.4	86.3	45.5	63.2	PG 58-xx
		64	19.5	87.3	19.5		
	10	58	14.5	85.0	14.5	66.5	PG 64-xx
		64	29.2	84.5	29.3		
